# Editorial: Crosstalk Between the Metabolic and Cardiovascular Systems in the Brain

**DOI:** 10.3389/fphys.2021.834637

**Published:** 2022-01-27

**Authors:** Jin Kwon Jeong, Hai-Bin Ruan, Chitoku Toda

**Affiliations:** ^1^Department of Pharmacology and Physiology, School of Medicine and Health Sciences, The George Washington University, Washington, DC, United States; ^2^Department of Integrative Biology and Physiology, University of Minnesota Medical School, Minneapolis, MN, United States; ^3^Laboratory of Biochemistry, Graduate School of Veterinary Medicine, Hokkaido University, Sapporo, Japan

**Keywords:** metabolic syndrome, (pro)renin receptor, vascular endothelial growth factor, cardiometabolism, CNS

In human patients with hypertension, existing therapeutic treatments show a low successful rate to control blood pressure. This is mainly because cardiovascular problems are closely associated with other physiological parameters, including metabolic complications. Indeed, metabolic syndrome, a world spreading health concern, includes metabolic disorders, such as obesity, hyperinsulinemia, insulin resistance, and diabetes, as well as cardiovascular problems including stroke and hypertension. Therefore, the metabolic and cardiovascular systems are reciprocally associated with each other, and impairment in one system can directly affect pathophysiological development on the other side.

Needless to say, understanding of where and how reciprocal interaction between the metabolic and cardiovascular systems occurs is critical to develop better treatment for metabolic syndrome. In this regard, accumulating investigations have pointed a role for the central nervous system (CNS) in mediating functional crosstalk between the metabolic and cardiovascular signaling molecules, and further an implication of the CNS mechanisms in normal cardio-metabolic homeostasis as well as in the development of the metabolic syndrome. A focus of the proposed Research Topic entitled “Crosstalk between the metabolic and cardiovascular systems in the brain” was to cover a broad spectrum of biomedical research within the CNS, from molecular level to local and regional networks, involved in the cardio-metabolic homeostasis, and this Research Topic consists of two original research articles and four reviews.

A key feature of essential hypertension is neurohumoral dysfunction, characterized by elevations in sympathetic outflow and hormonal imbalance. For example, a chronic overactivation of the renin-angiotensin system (RAS), characterized by elevations of angiotensin-II (Ang-II) in circulation, drives hypertension development. Importantly, the brain establishes intrinsic RAS system and an overactivation of the brain RAS system is also strongly implicated in the development of hypertension. Accumulated evidence have suggested that a local production of brain Ang-II can be initiated by the (pro)renin receptor (PRR) that is abundantly present in multiple cardiometabolic brain regions, including the hypothalamic paraventricular nucleus (PVN) and rostral ventrolateral medulla (RVLM). However, the molecular and clinical evidence of the PRR in these brain nuclei in relation to cardiovascular diseases in humans have not been characterized yet. Studies by Mohsin et al. in this Research Topic addressed this question using the postmortem brain samples from normotensive and hypertensive humans. What they found using an immunohistochemical approaches is that the expression of PPR in both the PVN and RVLM was (1) exclusively observed only in neuronal populations; (2) increased with hypertension; and (3) positively correlated with age and systolic blood pressure. These molecular and anatomical findings strongly suggest an active involvement of the brain PRR mechanisms in the development of pathophysiological cardiovascular conditions, such as hypertension, in humans.

One of the most life-threatening complications in metabolic syndrome is stroke. After cerebral ischemia occurs, the brain tries increasing angiogenesis to get enough blood supply to survive. Vascular endothelial growth factor (VEGF) is an important regulator for angiogenesis after stroke. However, an overdose of VEGF expression increases the permeability of the blood-brain barrier, which leads to edema and neuroinflammation. Therefore, an elaborate control of VEGF expression is needed for brain survival. The article by Zhang et al. reports the molecular mechanism of how VEGF expression level is regulated appropriately after oxygen-glucose deprivation (OGD), a model of stroke. They found that OGD increases tube formation of brain microvascular endothelial cells (BMVECs) by increasing MYB expression, which is a transcriptional factor to regulate angiogenesis. They also found that MYB induces miR-150 expression, which is an inhibitory regulator of VEGF, and miR-150 also suppresses MYB expression as a negative feedback loop in BMVECs after OGD. The data suggests that the feedback loop between MYB and miR-150 may achieve the fine-tuning of VEGF expression and thus affects the pathophysiological process of stroke.

Including these two research articles as mentioned above, all articles presented in this Research Topic represent a central role of the CNS mechanisms in cardio-metabolic physiology, by mediating reciprocal communication between the metabolic and cardiovascular signaling molecules.

## Author Contributions

JJ and CT developed a draft of manuscript. All authors have equally contributed to modify and approved it for publication.

## Conflict of Interest

The authors declare that the research was conducted in the absence of any commercial or financial relationships that could be construed as a potential conflict of interest.

## Publisher's Note

All claims expressed in this article are solely those of the authors and do not necessarily represent those of their affiliated organizations, or those of the publisher, the editors and the reviewers. Any product that may be evaluated in this article, or claim that may be made by its manufacturer, is not guaranteed or endorsed by the publisher.

